# Synthesis and Biological Evaluation of New Antitubulin Agents Containing 2-(3′,4′,5′-trimethoxyanilino)-3,6-disubstituted-4,5,6,7-tetrahydrothieno[2,3-*c*]pyridine Scaffold

**DOI:** 10.3390/molecules25071690

**Published:** 2020-04-07

**Authors:** Romeo Romagnoli, Filippo Prencipe, Paola Oliva, Barbara Cacciari, Jan Balzarini, Sandra Liekens, Ernest Hamel, Andrea Brancale, Salvatore Ferla, Stefano Manfredini, Matteo Zurlo, Alessia Finotti, Roberto Gambari

**Affiliations:** 1 Dipartimento di Scienze Chimiche e Farmaceutiche, University of Ferrara, Via L. Borsari 46, 44121 Ferrara, Italy; prnfpp@unife.it (F.P.); lvopla@unife.it (P.O.); ccb@unife.it (B.C.); 2 Laboratory of Virology and Chemotherapy, Rega Institute for Medical Research, KU Leuven, B-3000 Leuven, Belgium; jan.balzarini@rega.kuleuven.be (J.B.); sandra.liekens@kuleuven.be (S.L.); 3 Screening Technologies Branch, Developmental for Cancer Research, National Cancer Institute, National Institute of Health, Frederick, MD 21702, USA; hamele@dc37a.nci.nih.gov; 4 School of Pharmacy and Pharmaceutical Sciences, Cardiff University, King Edward VII Avenue, Cardiff CF10 3NB, UK; brancalea@cardiff.ac.uk (A.B.); ferlas1@cardiff.ac.uk (S.F.); 5 Dipartimento di Scienze della Vita e Biotecnologie, Università di Ferrara, 44121 Ferrara, Italy; stefano.manfredini@unife.it (S.M.); matteo.zurlo@unife.it (M.Z.); alessia.finotti@unife.it (A.F.); roberto.gambari@unife.it (R.G.); 6 Interuniversity Consortium for Biotechnology, Area di Ricerca, Padriciano, 34149 Trieste, Italy

**Keywords:** microtubules, structure–activity relationship, antiproliferative activity, tubulin, tetrahydrothieno[2,3-*c*]pyridine

## Abstract

Two novel series of compounds based on the 4,5,6,7-tetrahydrothieno[2,3-*c*]pyridine and 4,5,6,7-tetrahydrobenzo[*b*]thiophene molecular skeleton, characterized by the presence of a 3′,4′,5′-trimethoxyanilino moiety and a cyano or an alkoxycarbonyl group at its 2- or 3-position, respectively, were designed, synthesized, and evaluated for antiproliferative activity on a panel of cancer cell lines and for selected highly active compounds, inhibition of tubulin polymerization, and cell cycle effects. We have identified the 2-(3′,4′,5′-trimethoxyanilino)-3-cyano-6-methoxycarbonyl-4,5,6,7-tetrahydrothieno[2,3-*c*]pyridine derivative **3a** and its 6-ethoxycarbonyl homologue **3b** as new antiproliferative agents that inhibit cancer cell growth with IC_50_ values ranging from 1.1 to 4.7 μM against a panel of three cancer cell lines. Their interaction with tubulin at micromolar levels leads to the accumulation of cells in the G2/M phase of the cell cycle and to an apoptotic cell death. The cell apoptosis study found that compounds **3a** and **3b** were very effective in the induction of apoptosis in a dose-dependent manner. These two derivatives did not induce cell death in normal human peripheral blood mononuclear cells, suggesting that they may be selective against cancer cells. Molecular docking studies confirmed that the inhibitory activity of these molecules on tubulin polymerization derived from binding to the colchicine site.

## 1. Introduction

Cancer, which is the result of deviation of cell growth control mechanisms, has become a major worldwide disease and the second leading cause of mortality in developed countries, with almost 20 million deaths annually [1,2]. Many of the current treatments, involving an integrated treatment of surgery, radiation, and chemotherapy, have problems with toxicity and drug resistance [3,4]. Therefore, there is a strong demand for the discovery and development of effective chemotherapeutic agents with high selectivity and low toxicity [5,6,7].

The central role of microtubules in cell division and mitosis makes them an important target for the development of potential new anticancer agents [8,9,10]. A large number of small molecules displaying wide structural diversity and derived from natural sources or obtained by chemical synthesis have been identified within the last several decades [11,12,13] and have been shown to interfere with microtubules, polymeric structures composed of α- and β-tubulin heterodimers [14,15,16].

Prominent examples of tubulin-binding compounds used clinically for cancer treatment include microtubule-stabilizers that inhibit microtubule function by promoting abnormally high levels of tubulin polymerization. Examples of such drugs include paclitaxel (Taxol) and its semisynthetic analogue docetaxel (Taxotere) [17,18]. In contrast, the vinca alkaloids vincristine, vinorelbine, and vinblastine destabilize microtubules and act as microtubule depolymerizers by inhibiting tubulin polymerization [19]. Both taxoids and vinca alkaloids interact with β-tubulin, but at different sites, named the taxol and vinca sites, respectively [20]. The colchicine-site binders that inhibit tubulin assembly [21] are another class of compounds in discovery and development, including combretastatin A-4 phosphate (CA-4P) [22], that bind to the colchicine site located between the α- and β-tubulin subunits in the heterodimer, distinct from the vinca site located between adjacent α- and β-tubulin heterodimers [23].

Although many chemically diverse synthetic tubulin inhibitors have been synthesized, there is still a need to identify novel small molecules that target microtubules [24,25,26,27,28]. Among such compounds, thiophene [29,30,31,32,33,34] and fused thiophenes such as benzo[*b*]thiophenes [35,36,37,38,39,40], thieno[3,2-*d*]pyrimidines [41,42,43], and thieno[2,3-*b*]pyridines [44] were present as molecular skeletons in a significant number of synthetic inhibitors of microtubule polymerization, but there are limited examples of small molecules showing interesting activities in inhibiting both microtubule assembly and cell proliferation based on the 4,5,6,7-tetrahydrothieno[2,3-*c*]pyridine molecular skeleton as the core structure.

**Figure 1:**
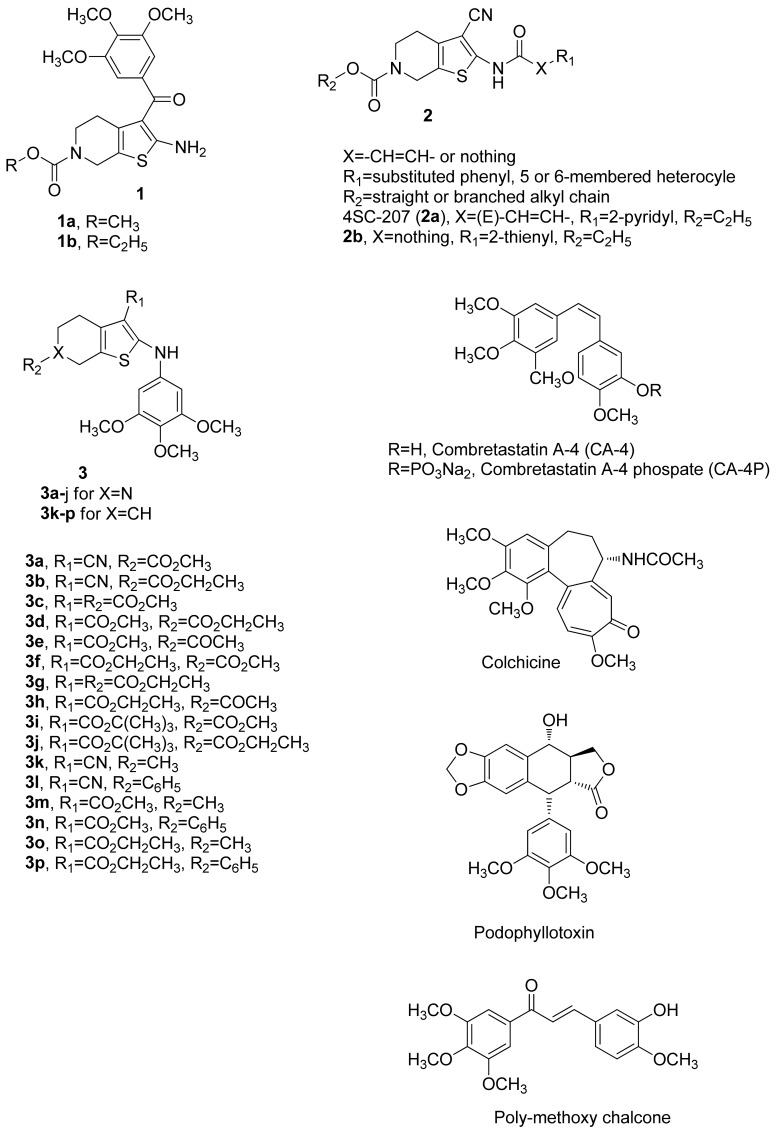
Structures of tetrahydrothieno[2,3-*c*]pyridines **1**–**3**. Compounds with a 3,4,5-trimethoxyphenyl moiety, such as colchicine, CA-4, podophyllotoxin, and poly-methoxychalcone.

In an earlier published study, a series of molecules with general structure **1** (Figure 1), based on the 2-amino-3-(3′,4′,5′-trimethoxybenzoyl)-4,5,6,7-tetrahydrothieno[2,3-*c*]pyridine skeleton, yielded the promising derivatives **1a** and **1b**. These compounds inhibit cancer cell growth with IC_50_ values ranging from 25 to 440 nM against a panel of four cancer cell lines and exert strong inhibition of tubulin polymerization by binding to the colchicine site of tubulin [45]. For this series of molecules, the presence of a *N*-methoxy/ethoxycarbonyl moiety at the 6-position of the 4,5,6,7-tetrahydrothieno[2,3-*c*]pyridine system was needed for potent antiproliferative activity.

A second series of compounds with general structure **2**, reported by Beckers et al. [46,47], incorporated the 2-carboxamido-3-cyano-4,5,6,7-tetrahydrothieno[2,3-*c*]pyridine system, and these agents were active at micromolar concentrations (IC_50_ = 0.2–5 μM) as antiproliferative agents against human colon adenocarcinoma (RKOp27) cells. Bausch et al. [48] reported a molecule named 4SC-207 (**2a**), characterized by the presence of the 2-[(pyridin-3′-yl)acrylamido]-3-cyano-4,5,6,7-tetrahydrothieno[2,3-*c*]pyridine skeleton, able to inhibit microtubule polymerization at high concentrations and showing strong antiproliferative activity at low nanomolar concentrations in a large panel of cancer cell lines. These studies [46,47,48] showed that the presence of a cyano group at the 3-position of the 4,5,6,7-tetrahydrothieno[2,3-*c*]pyridine nucleus was required for antiproliferaive activity. These findings prompted us to analyze this class of compounds in more detail.

Building upon these observations, since the 6-*N*-alkoxycarbonyl-4,5,6,7-tetrahydrothieno[2,3-*c*]pyridine structure was demonstrated to be essential for bioactivity, we retained this pharmacophoric unit throughout the present investigation and kept the 3-cyano group in compounds we synthesized. We were intrigued by the idea of studying the biological effects of replacing the carboxamido group at the 2-position of the 4,5,6,7-tetrahydrothieno[2,3-*c*]pyridine ring of compounds with general structure **2** with a 3′,4′,5′-trimethoxyanilino moiety to yield the 2-(3′,4′,5′-trimethoxyanilino)-3-cyano-6-methoxy/ethoxycarbonyl-4,5,6,7-tetrahydrothieno[2,3-*c*]pyridines **3a** and **3b**. Previous studies have shown that the 3,4,5-trimethoxyphenyl moiety is a pharmacophoric group that is an essential structural requirement for the anticancer effect of numerous inhibitors of tubulin polymerization, such as colchicine, combretastatin A-4 (CA-4), podophyllotoxin, and poly-methoxychalcone [49,50]. Starting from compounds **3a** and **3b**, additional analogues were also generated by modification of the 3-cyano function of the 4,5,6,7-tetrahydrothieno[2,3-*c*]pyridine skeleton. The cyano group was replaced with a 3-alkoxycarbonyl moiety, such as 3-methoxycarbonyl (**3c**–**3e**), 3-ethoxycarbonyl (**3f**–**3h**), or 3-*tert*-butoxycarbonyl (**3i**–**3j**). Moreover, the influence on antiproliferative activity of the substituent (R_2_) at the 6-position was evaluated with R_2_ as acetyl or methoxy/ethoxycarbonyl.

Additional structural modifications involved the nitrogen at the 6-position of 4,5,6,7-tetrahydrothieno[2,3-*c*]pyridine scaffold, by the classical bioisosteric replacement with a CH group to furnish a novel series of 2-(3′,4′,5′-trimethoxyanilino)-3,6-substituted-4,5,6,7-tetrahydrobenzo[*b*]thiophene derivatives **3k**–**3p**. For this latter series of compounds, besides the C-3 cyan and methoxy/ethoxycarbonyl moieties, the substituents examined at the C-6 position were methyl (**3k**, **3m**, and **3o**) and phenyl (**3l**, **3n**, and **3p**) groups.

## 2. Chemistry

**Scheme 1:**
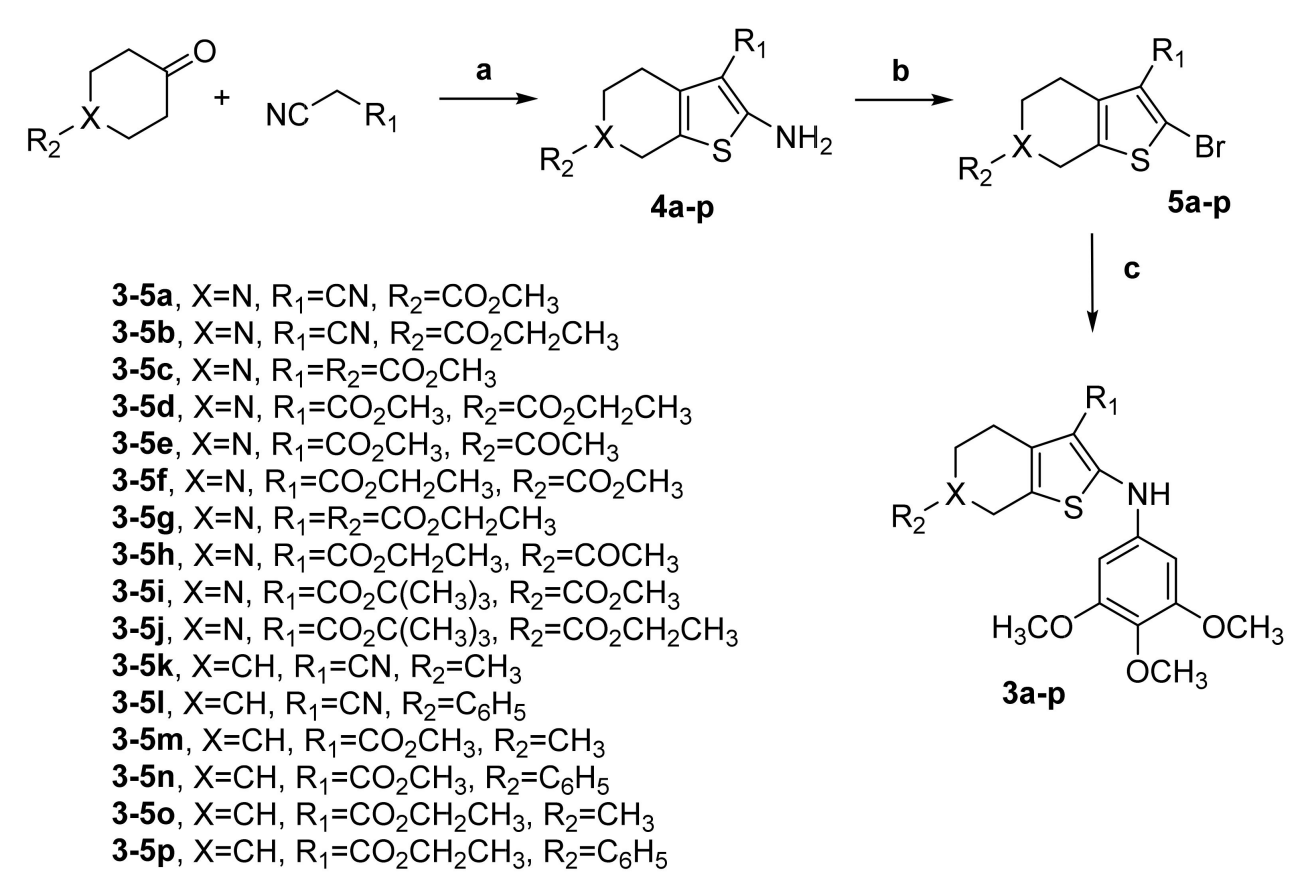
Synthesis of compounds **3a-p.** Reagents. **a**: appropriate ketone (4-methyl/phenylcyclohexanone, *N*-acetyl-4-piperidone, or methoxy/ethoxycarbonyl-4-piperidone), malonitrile or methyl/ethyl/*t*-butylcyanoacetate, S_8_, TEA, MeOH or EtOH, reflux; **b**: *t*-BuONO, CuBr_2_, CH_3_CN, 65 °C; **c**: 3,4,5-trimethoxyaniline, Pd(OAc)_2_, BINAP, Cs_2_CO_3_, PhMe, 100 °C, 18 h.

The 2-(3′,4′,5′-trimethoxyanilino)-3-cyano/alkoxycarbonyl-6-substituted-4,5,6,7-tetrahydrothieno[2,3-*c*]pyridines **3a**–**3j** and the corresponding 4,5,6,7-tetrahydrobenzo[*b*]thiophenes **3k**–**3p** were prepared through the three-step procedure shown in Scheme 1.

The one-pot Gewald reaction [51] applied to commercially available 4-methyl/phenylcyclohexanone, *N*-acetyl-4-piperidone or methoxy/ethoxycarbonyl-4-piperidone with an activated nitrile (malonitrile or a cyanoacetate, such as methyl/ethyl/*t*-butylcyanoacetate) and elemental sulfur in refluxing methanol or ethanol in the presence of triethylamine (TEA) as base furnished 2-amino-3-cyano/alkoxycarbonyl-6-substituted-4,5,6,7-tetrahydrothieno[2,3-*c*]pyridines **4a**–**4j** and the related 4,5,6,7-tetrahydrobenzo[*b*]thiophene analogues **4k**–**4p**. These latter compounds were transformed by substitutive deamination with *tert*-butyl nitrite (*t*-BuONO) and copper (II) bromide (CuBr_2_) in acetonitrile into the 2-bromo-4,5,6,7-tetrahydrothieno[2,3-*c*]pyridines **5a**–**5j** and 4,5,6,7-tetrahydrobenzo[*b*]thiophene analogues **5k**–**5p**, respectively [52]. Finally, the novel 2-(3′,4′,5′-trimethoxyanilino)-4,5,6,7-tetrahydrothieno[2,3-*c*]pyridines **3a**–**3j** and 2-(3′,4′,5′-trimethoxyanilino)-4,5,6,7-tetrahydrobenzo[*b*]thiophenes **3k**–**3p** were prepared in good yields by palladium-catalyzed arylamination conditions of the appropriate 2-bromo-4,5,6,7-tetrahydrothieno[2,3-*c*]pyridine **5a-j** and 2-bromo-4,5,6,7-tetrahydrobenzo[b]thiophene analogues **5k**–**5p**, respectively, with 3,4,5-trimethoxyaniline in the presence of palladium (II) acetate (Pd(OAc)_2_), *rac*-2,2′-bis-(diphenylphosphane)-1,1′-binaphtyl (BINAP), and cesium carbonate (Cs_2_CO_3_) (as catalyst, ligand, and base, respectively) in toluene [53].

## 3. Results and Discussion

### 3.1. In Vitro Antiproliferative Activities Against A Panel of Three Different Cancer Cell Lines (L1210, CEM, and HeLa)

In Table 1 we report the in vitro antiproliferative activity of 2-(3′,4′,5′-trimethoxyanilino)-4,5,6,7-tetrahydrothieno[2,3-*c*]pyridine derivatives **3a**–**3j** and the related 4,5,6,7-tetrahydrobenzo[*b*]thiophenes **3k**–**3p** against a panel of three cell lines, which were derived from different cancer types, including murine leukemia (L1210), human T-lymphoblastoid leukemia (CEM), and human cervix carcinoma (HeLa) cells. CA-4 and 2-carboxamido-3-cyano-6-ethoxycarbonyl-4,5,6,7-tetrahydrothieno[2,3-*c*]pyridine **2b** used in Beckers’ study [46,47] were also used as positive controls. All compounds that had an IC_50_ > 20 μM are considered inactive for purposes of this discussion.

Compound **3b** with an ethoxycarbonyl substituent at the C-6 position of the 2-(3′,4′,5′-trimethoxyanilino)-3-cyano-4,5,6,7-tetrahydrothieno[2,3-*c*]pyridine system, along with its 6-methoxycarbonyl analogue **3a,** exhibited the greatest antiproliferative activity among the tested compounds, with IC_50_ values of 1.1–2.8 and 1.9–4.7 μM, respectively, showing improved activity against HeLa cells as compared with the other two cell lines. The 3-cyano-6-ethoxycarbonyl-4,5,6,7-tetrahydrothieno[2,3-*c*]pyridine derivative **3b** possessed the highest potency, inhibiting the growth of L1210, CEM, and HeLa cells with IC_50_ values of 2.8, 2.3, and 1.1 μM, respectively. These values are 1.5-fold lower than those obtained with the 6-methoxycarbonyl derivative **3a** against L1210 and HeLa cells, while both compounds were equipotent against CEM cells. The activities of 2-(3′,4′.5′-trimethoxyanilino) derivatives **3a** and **3b** were quite similar to that of 2-(thiophene-2′-carboxamido) analogue **2b** against L1210 and CEM cells, while there was greater difference with the HeLa cell line, with compounds **3a** and **3b** 40- and 20-fold more active than **2b** (IC_50_: 41 μM). However, CA-4 was more potent than any of the new compounds in all cell lines examined. The activity of the most effective compounds (**3a** and **3b**) was also evaluated on the human chronic myelogenous leukemia K562 cell line. The data obtained demonstrated that both **3a** and **3b** were highly efficient in inhibiting K562 cells, displaying IC_50_ values of 0.75 and 0.70 μM, respectively.

The C-3-position of the 2-(3′,4′,5′-trimethoxyanilino)-4,5,6,7-tetrahydrothieno[2,3-*c*]pyridine nucleus plays an essential role in the antiproliferative activity of derivatives **3a**–**3j**. The activity of compounds **3a** and **3b** demonstrated that the 3-cyano substitution was crucial for potent cell growth inhibition. Its substitution with an alkoxycarbonyl moiety led to a reduction of growth inhibition activity (compare **3a** with **3c**, **3f**, and **3i** as well as **3b** with **3d**, **3g**, and **3j**). For the most active compound of the series (**3b**), replacement of the 3-cyano group with alkoxycarbonyl moieties, such as methoxycarbonyl, ethoxycarbonyl or *tert*-butoxycarbonyl (compounds **3d**, **3g**, and **3j**, respectively) reduced antiproliferative activity from 5- to 18-fold against the three cancer cell lines.

Finally, although only a few compounds were synthesized thus far in the 4,5,6,7-tetrahydrobenzo[*b*]thophene series, all compounds of this series proved inactive (IC_50_ >50 μM), significantly less potent than the corresponding 4,5,6,7-tetrahydrothieno[2,3-*c*]pyridine derivatives. The reduced antiproliferative activity of the 4,5,6,7-tetrahydrobenzo[*b*]thiophene derivatives as compared with the 4,5,6,7-tetrahydrothieno[2,3-*c*]pyridine compounds demonstrates that the presence of a piperidine ring fused with the thiophene scaffold is critical for activity.

In order to investigate the selective antiproliferative activities of derivatives **3a** and **3b** in normal human cells, both these molecules were tested in vitro against human peripheral blood mononuclear cells (PBMC) isolated from healthy donors. After treatment of both resting and phytohemagglutin (PHA)-stimulated PBMC for 72 h, the IC_50_ values were over 20 µM, indicating that **3a** and **3b** did not substantially affect the viability of these cells, suggesting that these two compounds have cancer cell selective killing properties.

### 3.2. Inhibition of Tubulin Polymerization and Colchicine Binding

To investigate whether the antiproliferative activities of these compounds were related to an interaction with the microtubule system, the two most active compounds of the series (**3a** and **3b**) as antiproliferative agents were evaluated for their inhibitory effects on tubulin polymerization and on the binding of [^3^H]colchicine to tubulin (in the latter assay, tubulin was examined at a concentration of 1 μM, while compounds and colchicine were at 5 μM). For comparison, CA-4 was examined in contemporaneous experiments as a reference compound (Table 2). Derivatives **3a** and **3b** inhibited the assembly reaction with IC_50_ values of 3.6 and 3.8 μM, respectively, considerably higher than the value obtained with CA-4 (IC_50_: 0.54 μM), which is consistent with the reduced antiproliferative activity of **3a** and **3b** with respect to CA-4. These results indicated that tubulin may be the intracellular target of compounds **3a** and **3b**. In competition experiments, both these compounds inhibited the binding of [^3^H]colchicine to its binding site on tubulin, with 31% and 29% of inhibition, respectively. These derivatives were less potent than CA-4, which in these experiments inhibited colchicine binding by 98%.

The results are consistent with the conclusion that the antiproliferative activity of these compounds derives from an interaction with the colchicine site of tubulin and interference with microtubule assembly. In conclusion, manipulation of the scaffold of compounds with general structures **1** and **2** led to the successful identification of a new series of inhibitors of tubulin assembly, characterized by a 3,4,5-trimethoxy aniline and a cyano function at the 2- and 3-positions, respectively, of the 4,5,6,7-tetrahydrothieno[2,3-*c*]pyridine nucleus.

### 3.3. Analysis of Effects on the Cell Cycle

**Figure 2:**
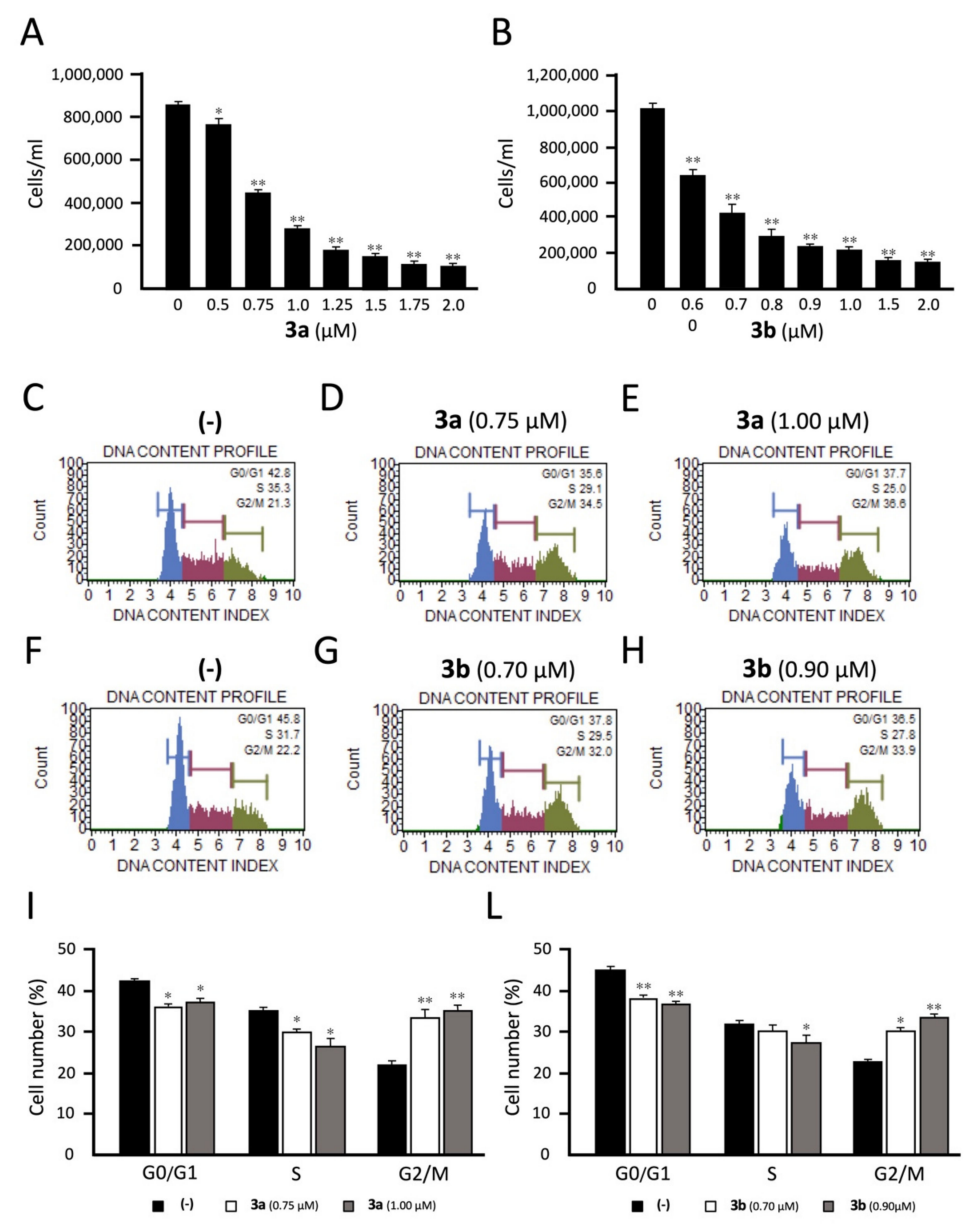
Effects of the tetrahydrothieno[2,3-*c*]pyridines **3a** and **3b** on cell growth and cell cycle. K562 cells were treated with compounds **3a** (**A,C**–**E,I**) and 3b (**B,F**–**H,L**) and the following biological parameters were evaluated: cell growth (**A,B**) and cell cycle distribution (**C**–**H**). Analyses were performed after 3 days of cell culture at the indicated concentrations of the compounds. In panels C-H, representative cell cycle analyses are reported. In panels I and L, a summary of three independent experiments is reported. The data represent the average ± S.D. *: significant (*p* < 0.05); **: highly significant (*p* < 0.01).

Since molecules exhibiting effects on tubulin assembly should cause alteration of cell cycle parameters, leading to a preferential G2/M blockade, the effects of compounds **3a** and **3b** on cell cycle distribution were investigated on K562 cells using flow cytometry. The cells were cultured for 72 h in the presence of two different concentrations (IC_50_ and IC_75_) for each tested derivative and the obtained results were compared with non-treated K562 cells as control. Both compounds caused a significant (*p* < 0.01) and dose-dependent accumulation of the cells in the G2/M phase of the cell cycle, with a concomitant decrease of cells in the other phases of the cell cycle (especially the G0/G1 phase). As shown in Figure 2 (panels I and L), treatment of K562 cells at different concentrations of **3b** (IC_50_: 0.70 μM and IC_75_: 0.90 μM) increased the percentage of G2/M-phase cells from 22.9% ± 0.7% (control group) to 30.3% ± 1.3% and 33.5% ± 0.6% respectively, indicating that compound **3b** impact the cell cycle distribution in a dose-dependent manner. Similarly, for derivative **3a** (IC_50_: 0.75 μM and IC_75_: 1.0 μM), an increase of the percentage of G2/M-phase cells from 22.2% ± 0.7% (control group) to 33.6% ± 1.1%, and 34.9% ± 1.3% was observed at the values, respectively. These data confirm that compounds **3a** and **3b** act selectively on the G2-M phase of the cell cycle, as expected for inhibitors of tubulin assembly.

### 3.4. Effects on Apoptosis

**Figure 3:**
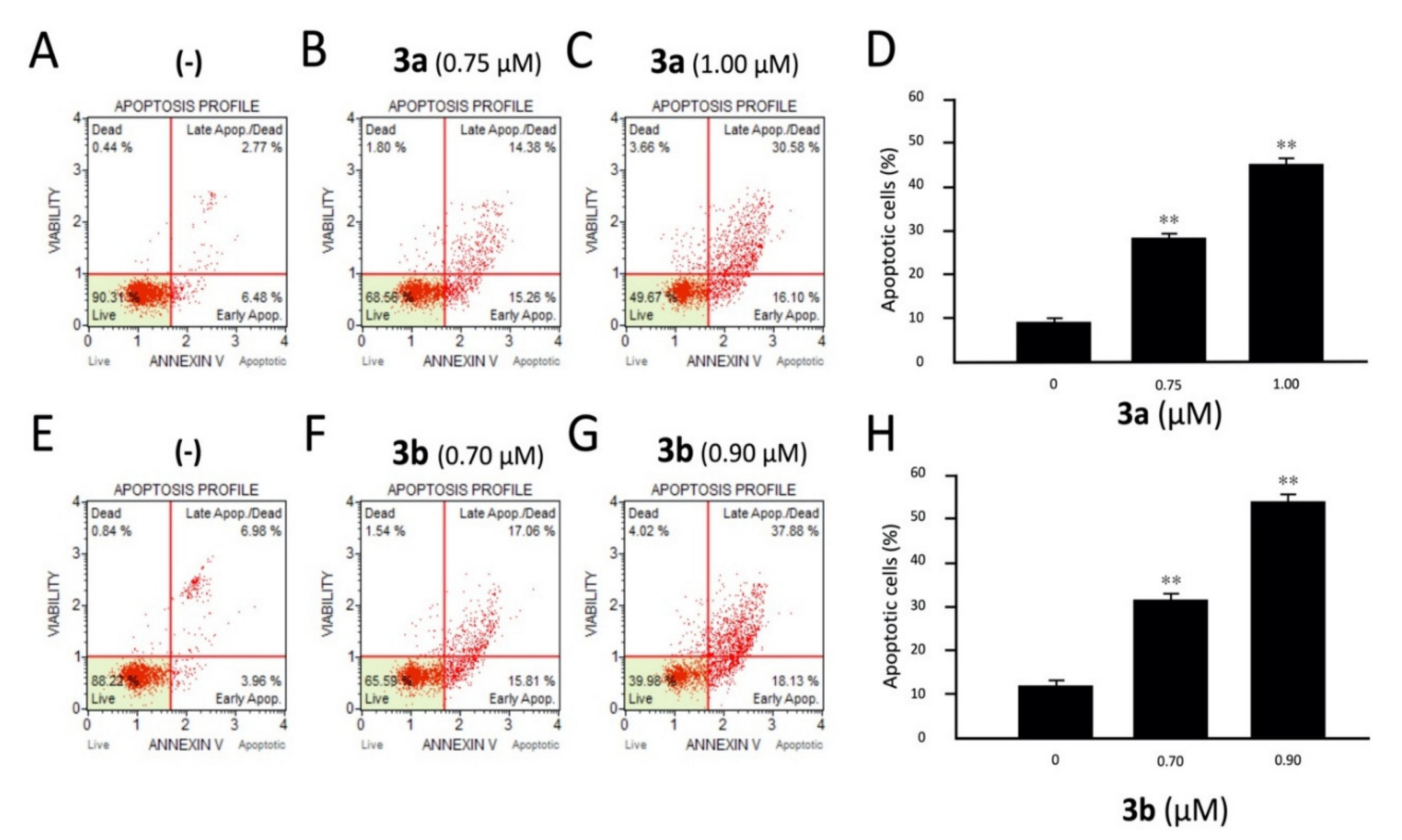
Effects of the tetrahydrothieno[2,3-*c*]pyridines **3a** and **3b** on apoptosis. K562 cells were treated with compounds **3a** (**A**–**D**) and **3b** (**E**–**H**), and apoptosis was analyzed by cytofluometry. Analyses were performed after 3 days of cell culture at the indicated concentrations of **3a** and **3b** compounds. In panels **A**–**C** and **E**–**G**, representative analyses of apoptosis are reported for compound **3a** (**A**–**C**) and compound 3b (**E**–**G**). In panels D and H, a summary of three independent experiments is presented. The data represent the average ± S.D. **: highly significant (*p* < 0.01).

In order to characterize the mode of cell death induced by compounds **3a** and **3b**, a biparametric flow cytometry analysis was performed using propidium iodide (PI), which stains DNA and is permeable only to dead cells, and fluorescent immunolabeling of the protein annexin-V, which binds to the phospholipid phosphatidylserine (PS) in a highly selective manner. This phospholipid flips from the inner to the outer leaflet of the plasma membrane during apoptosis. Positive staining with annexin-V correlates with the loss of plasma membrane polarity, but this staining precedes the complete loss of membrane integrity that accompanies the later stages of cell death, resulting from either apoptosis or necrosis. In contrast, PI can only enter cells after complete loss of membrane integrity. Thus, dual staining for annexin-V and with PI permits discrimination between unaffected cells (annexin-V^−^/PI^−^), early apoptotic cells (annexin-V^+^/PI^−^), late apoptotic cells (annexin-V^+^/PI^+^), and necrotic cells (annexin-V^−^/PI^+^). The results obtained are shown in Figure 3.

The obtained two parameter histograms demonstrate the effects of different concentrations of **3a** (IC_50_: 0.75 μM and IC_75_: 1.00 μM) and **3b** (IC_50_: 0.70 μM and IC_75_: 0.90 μM) on K562 cells after 72 h of treatment. Both compounds induced an accumulation of annexin-V positive cells in comparison with the control, and this accumulation was dose dependent. In the representative experiment shown in Figure 3, the amount of total apoptotic cells did not exceed 11% in the negative controls (not treated samples). On the contrary, compound **3b** at the IC_50_ (0.70 μM) and IC_75_ (0.90 μM) values after 72 h of treatment showed 32.87% and 56.01% cells undergoing apoptosis, respectively. Similarly, **3a** is also very effective in the induction of apoptosis in a dose-dependent manner, showing 29.64% and 46.68% cells in apoptotic phase at its IC_50_ (0.75 μM) and IC_75_ (1.00 μM) values, respectively. The results indicated that most of K562 cells treated with **3a** and **3b** undergo apoptosis.

### 3.5. Molecular Modeling Studies

The potential interaction between compounds **3a** and **3b** and the colchicine site was investigated through molecular docking studies, using Glide. [54] The colchicine-tubulin complex (PDB ID: 4O2B) crystal structure was selected as the protein for the docking simulation. [55]

**Figure 4:**
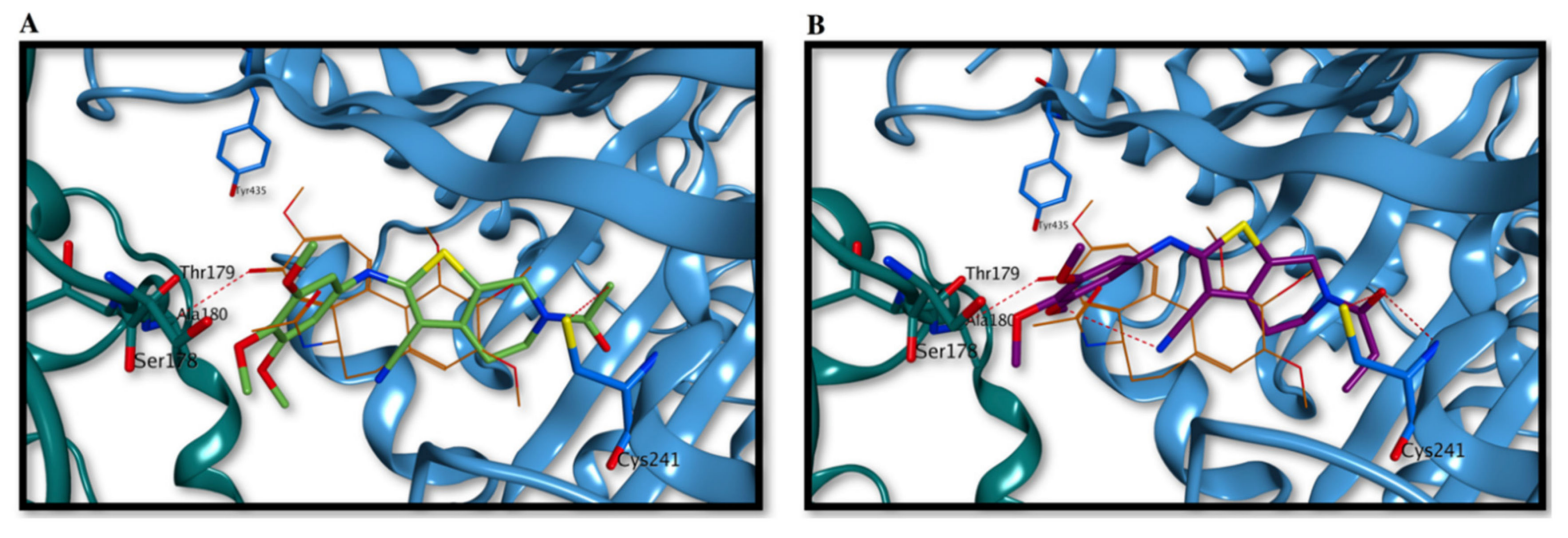
Proposed binding modes for compounds **3a** (**A**) and **3b** (**B**) in the colchicine site. The trimethoxyphenyl ring is oriented towards the α-tubulin subunit, while the *N*-methoxy/ethoxycarbonyl moiety at the 6-position of the tetrahydrothieno[2,3-*c*]pyridine ring is placed in proximity of βCys241. Co-crystallized colchicine is shown in orange. The tubulin α subunit is shown as a dark green ribbon, while the β subunit is represented as a cornflower blue ribbon.

Both compounds seem to occupy the binding site partially overlapping the co-crystallized colchicine, with the trimethoxyphenyl ring orientated towards the nearby α-tubulin subunit, with interactions αSer178 and αThr179 (Figure 4A,B). The *N*-methoxy/ethoxycarbonyl moiety at the 6-position of the tetrahydrothieno[2,3-*c*]pyridine ring is placed in proximity and interacts with either the polypeptide backbone of β-tubulin or with the sidechain of βCys241, a key interaction point for tubulin polymerization inhibition. Interestingly, the two molecules adopt a different orientation than the canonical binding previously reported for several tubulin inhibitors, including combretastatin A-4, in which the trimethoxyphenyl ring is placed in the β-tubulin subunit in proximity to βCys241 [56,57]. This occupation of the colchicine site seems to be fundamental for strong inhibition of tubulin polymerization, and therefore, the different orientation found for compounds **3a** and **3b** could explain their apparently weaker binding to tubulin and causing their reduced inhibitory effects on tubulin assembly/colchicine binding described above.

## 4. Materials and Methods

### 4.1. Chemistry

#### 4.1.1. Materials and Methods

^1^H experiments were recorded on either a Bruker AC 200 or a Varian 400 Mercury Plus spectrometer, while ^13^C-NMR spectra were recorded on a Varian 400 Mercury Plus spectrometer. Chemical shifts (δ) are given in ppm upfield, and the spectra were recorded in appropriate deuterated solvents, as indicated. Positive-ion electrospray ionization (ESI) mass spectra were recorded on a double-focusing Finnigan MAT 95 instrument with BE geometry. Melting points (mp) were determined on a Büchi-Tottoli apparatus and are uncorrected. All products reported showed ^1^H and ^13^C-NMR spectra in agreement with the assigned structures. The purity of tested compounds was determined by combustion elemental analyses conducted by the Microanalytical Laboratory of the Chemistry Department of the University of Ferrara with a Yanagimoto MT-5 CHN recorder elemental analyzer. All tested compounds yielded data consistent with a purity of at least 95% as compared with the theoretical values. All reactions were carried out under an inert atmosphere of dry nitrogen, unless otherwise indicated. Standard syringe techniques were used for transferring dry solvents. Reaction courses and product mixtures were routinely monitored by Thin Layer Chromatography (TLC) on silica gel (precoated F254 Merck plates), and compounds were visualized with aqueous KMnO4. Flash chromatography was performed using 230–400 mesh silica gel and the indicated solvent system. Organic solutions were dried over anhydrous Na_2_SO_4_.

#### 4.1.2. General Procedure A for the Synthesis of Compounds (**4a**–**4p)**

To a suspension of malonitrile or cyanoacetate (methyl/ethyl/*t*-butylcyanoacetate) (5 mmol), the appropriate ketone corresponding to 4-methyl/phenylcyclohexanone, *N*-acetyl-4-piperidone or methoxy/ethoxycarbonyl-4-piperidone (5 mmol), TEA (0.44 mL, 5 mmol) and sulfur (164 mg, 5 mmol) in the appropriate solvent (methanol or ethanol, 10 mL) was stirred for 2 h at 70 °C. The solvent was evaporated, and the residue was diluted with dichloromethane (15 mL). After washing with water (2 × 5 mL) and brine (5 mL), the organic layer was dried over anhydrous Na_2_SO_4_ and evaporated. The crude product was purified by column chromatography on silica gel or by crystallization from ethyl ether.

*Methyl 2-amino-3-cyano-4,5-dihydrothieno[2,3-c]pyridine-6(7H)-carboxylate* (**4a**). Following general procedure A, the crude residue obtained by the condensation between malononitrile and methyl 4-oxopiperidine-1-carboxylate in ethanol as solvent was purified by crystallization with ethyl ether to furnish **4a** as an orange solid. Yield: 87%, m.p. 131–133 °C. ^1^H-NMR (*d_6_*-DMSO) δ: 2.38–2.44 (m, 2H), 3.56–3.59 (m, 2H), 3.62 (s, 3H), 4.29 (s, 2H), 7.14 (bs, 2H). MS (ESI): [M + 1]^+^ = 238.3.

*Ethyl 2-amino-3-cyano-4,5-dihydrothieno[2,3-c]pyridine-6(7H)-carboxylate* (**4b**). Following general procedure A, the crude residue obtained by the condensation between malononitrile and ethyl 4-oxopiperidine-1-carboxylate in ethanol as solvent was purified by crystallization with ethyl ether to furnish **4b** as an orange solid. Yield: 87%, m.p. 171–174 °C. ^1^H–NMR (CDCl_3_) δ: 1.29 (t, *J* = 7.2 Hz, 3H), 2.77 (t, *J* = 5.8 Hz, 2H), 3.74 (t, *J* = 5.8 Hz, 2H), 4.21 (q, *J* = 7.2 Hz, 2H), 4.56 (s, 2H), 4.67 (bs, 2H). MS (ESI): [M + 1]^+^ = 252.3.

*Dimethyl 2–amino-4,5-dihydrothieno[2,3-c]pyridine-3,6(7H)-dicarboxylate* (**4c**). Following general procedure A, the crude residue obtained by the condensation between methyl 2-cyanoacetate and methyl 4-oxopiperidine-1-carboxylate in methanol as solvent was purified by crystallization with ethyl ether to furnish **4c** as a brown solid. Yield: 67%, m.p. 135–137 °C. ^1^H-NMR (CDCl_3_) δ: 2.80–2.82 (m, 2H), 3.66 (t, *J* = 6.0 Hz, 2H), 3.74 (s, 3H), 3.86 (s, 3H), 4.42 (s, 2H), 4.52 (bs, 2H). MS (ESI): [M + 1]^+^ = 271.3.

*6-Ethyl 3-methyl 2-amino-4,5-dihydrothieno[2,3-c]pyridine-3,6(7H)-dicarboxylate* (**4d**). Following general procedure A, the crude residue obtained by the condensation between methyl 2-cyanoacetate and ethyl 4-oxopiperidine-1-carboxylate in methanol as solvent was purified by crystallization with ethyl ether to furnish **4d** as a yellow solid. Yield: 84%, m.p. 152–154 °C. ^1^H-NMR (CDCl_3_) δ: 1.27 (t, *J* = 7.0 Hz, 3H), 2.81 (t, *J* = 6.0 Hz, 2H), 3.66 (t, *J* = 6.0 Hz, 2H), 3.90 (s, 3H), 4.11 (bs, 2H), 4.15 (q, *J* = 7.0 Hz, 2H), 4.42 (s, 2H). MS (ESI): [M + 1]^+^ = 285.4.

*Methyl 6-acetyl-2-amino-4,5,6,7-tetrahydrothieno[2,3-c]pyridine-3-carboxylate* (**4e**). Following general procedure A, the crude residue obtained by the condensation between methyl 2-cyanoacetate and 1-acetylpiperidin-4-one in methanol as solvent was purified by crystallization with ethyl ether to furnish **4e** as a yellow solid. Yield: 72%, m.p. 144–146 °C. ^1^H-NMR (CDCl_3_) δ: 2.24 (s, 3H), 2.87 (t, *J* = 6.0 Hz, 2H), 3.72 (t, *J* = 6.0 Hz, 2H), 3.80 (s, 3H), 4.51 (s, 2H), 5.14 (bs, 2H). MS (ESI): [M + 1]^+^ = 255.3.

*3-Ethyl 6-methyl 2-amino-4,5-dihydrothieno[2,3-c]pyridine-3,6(7H)-dicarboxylate* (**4f**). Following general procedure A, the crude residue obtained by the condensation between ethyl 2-cyanoacetate and methyl 4-oxopiperidine-1-carboxylate in ethanol as solvent was purified by crystallization with ethyl ether to furnish **4f** as a yellow solid. Yield: 64%, m.p. 151–153 °C. ^1^H-NMR (CDCl_3_) δ: 1.33 (t, *J* = 7.0 Hz, 3H), 2.80-2.82 (m, 2H), 3.66 (t, *J* = 6.0 Hz, 2H), 3.74 (s, 3H), 3.78 (bs, 2H), 4.24 (q, *J* = 7.0 Hz, 2H), 4.41 (s, 2H). MS (ESI): [M + 1]^+^ = 285.4.

*Diethyl 2-amino-4,5-dihydrothieno[2,3-c]pyridine-3,6(7H)-dicarboxylate* (**4g**). Following general procedure A, the crude residue obtained by the condensation between ethyl 2-cyanoacetate and ethyl 4-oxopiperidine-1-carboxylate in ethanol as solvent was purified by crystallization with ethyl ether to furnish **4g** as a yellow solid. Yield: 78%, m.p. 145–147 °C. ^1^H-NMR (*d_6_*-DMSO) δ: 1.15-1.28 (m, 6H), 2.64-2.67 (m, 2H), 3.55 (t, *J* = 6.0 Hz, 2H), 4.04 (q, *J* = 7.2 Hz, 2H), 4.16 (q, *J* = 7.2 Hz, 2H), 4.29 (s, 2H), 7.32 (bs, 2H). MS (ESI): [M + 1]^+^ = 299.1.

*Ethyl 6-acetyl-2-amino-4,5,6,7-tetrahydrothieno[2,3-c]pyridine-3-carboxylate* (**4h**). Following general procedure (A), the crude residue obtained by the condensation between ethyl 2-cyanoacetate and 1-acetylpiperidin-4-one in ethanol as solvent was purified by crystallization with ethyl ether to furnish **4h** as a yellow solid. Yield: 69%, m.p. 135–137 °C. ^1^H-NMR (CDCl_3_) δ: 1.36 (t, *J* = 7.4 Hz, 3H), 2.26 (s, 3H), 2.89 (t, *J* = 6.0 Hz, 2H), 3.73 (t, *J* = 6.0 Hz, 2H), 4.29 (q, *J* = 7.2 Hz, 2H), 4.38 (s, 2H), 4.96 (bs, 2H). MS (ESI): [M + 1]^+^ = 269.3.

*3-tert-butyl 6-methyl 2-amino-4,5-dihydrothieno[2,3-c]pyridine-3,6(7H)-dicarboxylate* (**4i**). Following general procedure A, the crude residue obtained by the condensation between methyl 2-cyanoacetate and *tert*-butyl 4-oxopiperidine-1-carboxylate in methanol as solvent was purified by crystallization with ethyl ether to furnish **4i** as a yellow solid. Yield: 78%, m.p. 134–136 °C. ^1^H-NMR (CDCl_3_) δ: 1.57 (s, 9H), 2.76–2.81 (m, 2H), 3.66–3.70 (m, 2H), 3.73 (s, 3H), 4.45 (s, 2H), 4.54 (bs, 2H). MS (ESI): [M + 1]^+^ = 313.4.

*3-tert-butyl 6-ethyl 2-amino-4,5-dihydrothieno[2,3-c]pyridine-3,6(7H)-dicarboxylate* (**4j**). Following general procedure A, the crude residue obtained by the condensation between ethyl 2-cyanoacetate and *tert*-butyl 4-oxopiperidine-1-carboxylate in ethanol as solvent was purified by crystallization with ethyl ether to furnish **4j** as a yellow solid. Yield: 84%, m.p. 152–154 °C. ^1^H-NMR (CDCl_3_) δ: 1.29 (t, *J* = 7.2 Hz, 3H), 1.55 (s, 9H), 2.78–2.81 (m, 2H), 3.66-3.69 (m, 2H), 4.19 (q, *J* = 7.2 Hz, 2H), 4.46 (s, 2H), 4.84 (bs, 2H). MS (ESI): [M + 1]^+^ = 327.4.

*2. -Amino-6-methyl-4,5,6,7-tetrahydrobenzo[b]thiophene-3-carbonitrile* (**4k**). Following general procedure A, the crude residue obtained by the condensation between malononitrile and 4-methylcyclohexanone in methanol as solvent was purified by trituration of the crude product with ethyl ether to furnish **4k** as a yellow solid. Yield: 58%, m.p. 124–126 °C. ^1^H-NMR (CDCl_3_) δ: 1.03 (d, *J* = 6.6 Hz, 3H), 1.39–1.42 (m, 1H), 1.84–1.88 (m, 2H), 2.08–2.12 (m, 1H), 2.56–2.60 (m, 3H), 4.29 (bs, 2H). MS (ESI): [M + 1]^+^ = 193.3.

*2. -Amino-6-phenyl-4,5,6,7-tetrahydrobenzo[b]thiophene-3-carbonitrile* (**4l**). Following general procedure (A), the crude residue obtained by the condensation between malononitrile and 4-phenylcyclohexanone in ethanol as solvent was purified by trituration of the crude product with ethyl ether to furnish **4l** as a yellow solid. Yield: 71%, m.p. 180–182 °C. ^1^H-NMR (CDCl_3_) δ: 1.19–1.22 (m, 1H), 2.07–2.10 (m, 1H), 2.66–2.71 (m, 3H), 2.72–2.76 (m, 2H), 2.97–3.00 (m, 1H), 3.39 (bs, 2H), 7.32 (m, 5H). MS (ESI): [M + 1]^+^ = 255.2.

*Methyl 2-amino-6-methyl-4,5,6,7-tetrahydrobenzo[b]thiophene-3-carboxylate* (**4m**). Following general procedure A, the crude residue obtained by the condensation between methyl 2-cyanoacetate and 4-methylcyclohexanone in methanol as solvent was purified by flash chromatography on silica gel, using ethyl acetate:petroleum ether 2:8 (v:v) as eluent, to furnish **4m** as a yellow solid. Yield: 65%, m.p. 109–110 °C. ^1^H-NMR (CDCl_3_) δ: 1.03 (d, *J* = 6.2 Hz, 3H), 1.34–1.38 (m, 1H), 1.84–1.88 (m, 2H), 2.15–2.19 (m, 1H), 2.63–2.66 (m, 2H), 2.84–2.87 (m, 1H), 3.88 (s, 3H), 4.64 (bs, 2H). MS (ESI): [M + 1]^+^ = 226.3.

*Methyl 2-amino-6-phenyl-4,5,6,7-tetrahydrobenzo[b]thiophene-3-carboxylate* (**4n**). Following general procedure A, the crude residue obtained by the condensation between methyl 2-cyanoacetate and 4-phenylcyclohexanone in methanol as solvent was purified by flash chromatography on silica gel, using ethyl acetate:petroleum ether 4:6 (*v*:*v*) as eluent, to furnish **4n** as a white solid. Yield: 62%, m.p. 132–134. °C ^1^H-NMR (CDCl_3_) δ: 1.80–1.84 (m, 1H), 2.02–2.06 (m, 1H), 2.75–2.79 (m, 3H), 2.99–3.02 (m, 2H), 3.86 (s, 3H), 4.82 (bs, 2H), 7.23–7.36 (m, 5H). MS (ESI): [M + 1]^+^ = 288.2.

*Ethyl 2-amino-6-methyl-4,5,6,7-tetrahydrobenzo[b]thiophene-3-carboxylate* (**4o**). Following general procedure A, the crude residue obtained by the condensation between ethyl 2-cyanoacetate and 4-methylcyclohexanone in ethanol as solvent was purified by flash chromatography on silica gel, using ethyl acetate:petroleum ether 2:8 (*v*:*v*) as eluent, to furnish **4o** as a yellow solid. Yield: 63%, m.p. 112–114. °C ^1^H-NMR (CDCl_3_) δ: 1.03 (d, *J* = 6.2 Hz, 3H), 1.29–1.32 (m, 1H), 1.35 (t, *J* = 7.0 Hz, 3H), 1.84–1.87 (m, 2H), 2.14–2.17 (m, 1H), 2.63–2.68 (m, 2H), 2.84–2.88 (m, 1H), 4.32 (q, *J* = 7.0 Hz, 2H), 5.82 (bs, 2H). MS (ESI): [M + 1]^+^ = 240.3.

*Ethyl 2-amino-6-phenyl-4,5,6,7-tetrahydrobenzo[b]thiophene-3-carboxylate* (**4p**). Following general procedure A, the crude residue obtained by the condensation between ethyl 2-cyanoacetate and 4-phenylcyclohexanone in ethanol as solvent was purified by flash chromatography on silica gel, using ethyl acetate:petroleum ether 4:6 (*v*:*v*) as eluent, to furnish **4p** as a yellow oil. Yield: 59%. ^1^H-NMR (CDCl_3_) δ: 1.34 (t, *J* = 7.0 Hz, 3H), 1.90–1.95 (m, 1H), 2.10–2.14 (m, 1H), 2.77–2.82 (m, 3H), 3.02–3.06 (m, 2H), 4.34 (q, *J* = 7.0 Hz, 2H), 4.76 (bs, 2H), 7.24–7.33 (m, 5H). MS (ESI): [M + 1]^+^ = 302.3.

#### 4.1.3. General Procedure B for the Synthesis of Compounds (**5a**–**p**)

In a dry three-necked round-bottom flask, anhydrous CuBr_2_ (536 mg, 2.4 mmol) and *tert*-butyl nitrite (360 μL, 3 mmol) were dissolved in anhydrous acetonitrile (10 mL) under an argon atmosphere. The resulting mixture was warmed at 65 °C and 2-amino derivative **4a**–**p** (2 mmol) in acetonitrile (5 mL) was slowly added. The reaction was complete after 2 h, as monitored by TLC. The dark mixture was allowed to reach room temperature, poured in a saturated aqueous NH_4_Cl solution (10 mL), and extracted with CH_2_Cl_2_ (30 mL). The organic phase was washed twice with a saturated aqueous NH_4_Cl solution (10 mL) and brine (10 mL), dried over Na_2_SO_4_, and concentrated at reduced pressure to furnish a residue that was purified by flash chromatography on silica gel.

*Methyl 2-bromo-3-cyano-4,5-dihydrothieno[2,3-c]pyridine-6(7H)-carboxylate* (**5a**). Following general procedure B, the crude residue was purified by flash chromatography, using ethyl acetate:petroleum ether 3:7 (*v*:*v*) as eluent, to furnish **5a** as a yellow solid. Yield: 64%, m.p. 153–155 °C. ^1^H-NMR (CDCl_3_) δ: 2.77 (t, *J* = 5.8 Hz, 2H), 3.71 (t, *J* = 5.8 Hz, 2H), 3.75 (s, 3H), 4.56 (s, 2H). MS (ESI): [M] ^+^ = 301.2, [M + 2]^+^ = 303.2.

*Ethyl 2-bromo-3-cyano-4,5-dihydrothieno[2,3-c]pyridine-6(7H)-carboxylate* (**5b**). Following general procedure B, the crude residue was purified by flash chromatography, using ethyl acetate:petroleum ether 3:7 (*v*:*v*) as eluent, to furnish **5b** as a white solid. Yield: 64%, m.p. 171–173 °C. ^1^H-NMR (CDCl_3_) δ: 1.28 (t, *J* = 7.0 Hz, 3H), 2.81 (t, *J* = 5.8 Hz, 2H), 3.73 (t, *J* = 5.8 Hz, 2H), 4.17 (q, *J* = 7.0 Hz, 2H), 4.66 (s, 2H). MS (ESI): [M]^+^ = 315.2, [M + 2]^+^ = 317.2.

*Dimethyl 2-bromo-4,5-dihydrothieno[2,3-c]pyridine-3,6(7H)-dicarboxylate* (**5c**). Following general procedure B, the crude residue was purified by flash chromatography, using ethyl acetate:petroleum ether 3:7 (*v*:*v*) as eluent, to furnish **5c** as a colorless oil. Yield: 64%. ^1^H-NMR (CDCl_3_) δ: 2.89 (t, *J* = 5.8 Hz, 2H), 3.68 (t, *J* = 5.8 Hz, 2H), 3.73 (s, 3H), 3.83 (s, 3H), 4.54 (s, 2H). MS (ESI): [M]^+^ = 334.1, [M + 2]^+^ = 336.3.

*6-Ethyl 3-methyl 2-bromo-4,5-dihydrothieno[2,3-c]pyridine-3,6(7H)-dicarboxylate* (**5d**). Following general procedure B, the crude residue was purified by flash chromatography, using ethyl acetate:petroleum ether 2:8 (*v*:*v*) as eluent, to furnish **5d** as a yellow oil. Yield: 73%. ^1^H-NMR (CDCl_3_) δ: 1.28 (t, *J* = 7.2 Hz, 3H), 2.89 (t, *J* = 6.0 Hz, 2H), 3.68 (t, *J* = 5.8 Hz, 2H), 3.87 (s, 3H), 4.16 (q, *J* = 7.2 Hz, 2H), 4.54 (s, 2H). MS (ESI): [M]^+^ = 348.3, [M + 2]^+^ = 350.3.

*Methyl 6-acetyl-2-bromo-4,5,6,7-tetrahydrothieno[2,3-c]pyridine-3-carboxylate* (**5e**). Following general procedure B, the crude residue was purified by flash chromatography, using ethyl acetate:petroleum ether 1:9 (*v*:*v*) as eluent, to furnish **5e** as a brown oil. Yield: 68%. ^1^H-NMR (CDCl_3_) δ: 2.21 (s, 3H), 3.05 (t, *J* = 6.0 Hz, 2H), 3.68 (t, *J* = 5.8 Hz, 2H), 3.83 (s, 3H), 4.76 (s, 2H). MS (ESI): [M]^+^ = 318.3, [M + 2]^+^ = 320.3.

*3-Ethyl 6-methyl 2-bromo-4,5-dihydrothieno[2,3-c]pyridine-3,6(7H)-dicarboxylate* (**5f**). Following general procedure B, the crude residue was purified by flash chromatography, using ethyl acetate:petroleum ether 2:8 (*v*:*v*) as eluent, to furnish **5f** as a yellow solid. Yield: 65%, m.p. 110–112 °C. ^1^H-NMR (CDCl_3_) δ: 1.32 (t, *J* = 7.2 Hz, 3H), 2.90 (t, *J* = 6.0 Hz, 2H), 3.68 (t, *J* = 5.8 Hz, 2H), 3.74 (s, 3H), 4.29 (q, *J* = 7.2 Hz, 2H), 4.55 (s, 2H). MS (ESI): [M]^+^ = 348.3, [M + 2]^+^ = 350.3.

*Diethyl 2-bromo-4,5-dihydrothieno[2,3-c]pyridine-3,6(7H)-dicarboxylate* (**5g**). Following general procedure B, the crude residue was purified by flash chromatography, using ethyl acetate:petroleum ether 2:8 (*v*:*v*) as eluent, to furnish **5g** as a yellow oil. Yield: 58%. ^1^H-NMR (CDCl_3_) δ: 1.28 (t, *J* = 7.0 Hz, 3H), 1.35 (t, *J* = 7.0 Hz, 3H), 2.86 (t, *J* = 5.6 Hz, 2H), 3.68 (t, *J* = 5.8 Hz, 2H), 4.16 (q, *J* = 7.2 Hz, 2H), 4.34 (q, *J* = 7.2 Hz, 2H), 4.54 (s, 2H). MS (ESI): [M]^+^ = 362.1, [M + 2]^+^ = 364.3.

*Ethyl 6-acetyl-2-bromo-4,5,6,7-tetrahydrothieno[2,3-c]pyridine-3-carboxylate* (**5h**). Following general procedure B, the crude residue was purified by flash chromatography, using ethyl acetate:petroleum ether 1:9 (*v*:*v*) as eluent, to furnish **5h** as a brown oil. Yield: 69%. ^1^H-NMR (CDCl_3_) δ: 1.35 (t, *J* = 7.2 Hz, 3H), 2.22 (s, 3H), 3.05 (t, *J* = 6.0 Hz, 2H), 3.72 (t, *J* = 5.8 Hz, 2H), 4,27 (q, *J* = 7.2 Hz, 2H), 4.76 (s, 2H). MS (ESI): [M]^+^ = 332.3, [M + 2]^+^ = 334.3.

*3-tert-butyl 6-methyl 2-bromo-4,5-dihydrothieno[2,3-c]pyridine-3,6(7H)-dicarboxylate* (**5i**). Following general procedure B, the crude residue was purified by flash chromatography, using ethyl acetate:petroleum ether 2:8 (*v*:*v*) as eluent, to furnish **5i** as a colorless oil. Yield: 63%. ^1^H-NMR (CDCl_3_) δ: 1.57 (s, 9H), 2.87 (t, *J* = 6.0 Hz, 2H), 3.67 (t, *J* = 5.8 Hz, 2H), 3.74 (s, 3H), 4.54 (s, 2H). MS (ESI): [M]^+^ = 376.3, [M + 2]^+^ = 378.3.

*3-tert-butyl 6-ethyl 2-bromo-4,5-dihydrothieno[2,3-c]pyridine-3,6(7H)-dicarboxylate* (**5j**). Following general procedure B, the crude residue was purified by flash chromatography, using ethyl acetate:petroleum ether 2:8 (*v*:*v*) as eluent, to furnish **5j** as a colorless oil. Yield: 71%. ^1^H-NMR (CDCl_3_) δ: 1.26 (t, *J* = 7.2 Hz, 3H), 1.55 (s, 9H), 2.87 (t, *J* = 6.0 Hz, 2H), 3.71 (t, *J* = 5.8 Hz, 2H), 4.16 (q, *J* = 7.2 Hz, 2H), 4.53 (s, 2H). MS (ESI): [M]^+^ = 390.3, [M + 2]^+^ = 392.3.

*2. -Bromo-6-methyl-4,5,6,7-tetrahydrobenzo[b]thiophene-3-carbonitrile* (**5k**). Following general procedure B, the crude residue was purified by flash chromatography, using ethyl acetate:petroleum ether 0.5:9.5 (*v*:*v*) as eluent, to furnish **5k** as a green oil. Yield: 72%. ^1^H-NMR (CDCl_3_) δ: 1.06 (d, *J* = 6.2 Hz, 3H), 1.26–1.28 (m, 1H), 1.89–1.92 (m, 2H), 2.20–2.24 (m, 1H), 2.69–2.72 (m, 3H). MS (ESI): [M]^+^ = 256.2, [M + 2]^+^ = 258.2.

*2. -Bromo-6-phenyl-4,5,6,7-tetrahydrobenzo[b]thiophene-3-carbonitrile* (**5l**). Following general procedure B, the crude residue was purified by flash chromatography, using ethyl acetate:petroleum ether 0.5:9.5 (*v*:*v*) as eluent, to furnish **5l** as a brown oil. Yield: 63%. ^1^H-NMR (CDCl_3_) δ: 1.80–1.83 (m, 1H), 2.17–2.20 (m, 1H), 2.75–2.79 (m, 3H), 2.98–3.01 (m, 2H), 7.26–7.31 (m, 5H). MS (ESI): [M]^+^ = 318.2, [M + 2]^+^ = 320.3.

*Methyl 2-bromo-6-methyl-4,5,6,7-tetrahydrobenzo[b]thiophene-3-carboxylate* (**5m**). Following general procedure B, the crude residue was purified by flash chromatography, using ethyl acetate:petroleum ether 0.5:9.5 (*v*:*v*) as eluent, to furnish **5m** as a colorless oil. Yield: 63%. ^1^H-NMR (CDCl_3_) δ: 1.06 (d, *J* = 6.6 Hz, 3H), 1.30–1.32 (m, 1H), 1.89-1.92 (m, 2H), 2.24–2.28 (m, 1H), 2.74-2.78 (m, 2H), 3.03-3.06 (m, 1H), 3.84 (s, 3H). MS (ESI): [M]^+^ = 289.2, [M + 2]^+^ = 291.2.

*Methyl 2-bromo-6-phenyl-4,5,6,7-tetrahydrobenzo[b]thiophene-3-carboxylate* (**5n**). Following general procedure B, the crude residue was purified by flash chromatography, using ethyl acetate:petroleum ether 0.5:9.5 (*v*:*v*) as eluent, to furnish **5n** as a colorless oil. Yield: 70%. ^1^H-NMR (CDCl_3_) δ: 1.77–1.82 (m, 1H), 2.08–2.10 (m, 1H), 2.78–2.82 (m, 3H), 3.00–3.04 (m, 2H), 3.84 (s, 3H), 7.24–7.30 (m, 5H). MS (ESI): [M]^+^ = 351.3, [M + 2]^+^ = 353.4.

*Ethyl 2-bromo-6-methyl-4,5,6,7-tetrahydrobenzo[b]thiophene-3-carboxylate* (**5o**). Following general procedure B, the crude residue was purified by flash chromatography, using ethyl acetate:petroleum ether 0.5:9.5 (*v*:*v*) as eluent, to furnish **5o** as a colorless oil. Yield: 72%. ^1^H-NMR (CDCl_3_) δ: 1.07 (d, *J* = 6.6 Hz, 3H), 1.31–1.34 (m, 1H), 1.37 (t, *J* = 7.0 Hz, 3H), 1.78-1.82 (m, 2H), 2.17–2.21 (m, 1H), 2.68–2.75 (m, 2H), 2.86–2.88 (m, 1H), 4.26 (q, *J* = 7.0 Hz, 2H). MS (ESI): [M]^+^ = 303.2, [M + 2]^+^ = 305.2.

*Ethyl 2-bromo-6-phenyl-4,5,6,7-tetrahydrobenzo[b]thiophene-3-carboxylate* (**5p**). Following general procedure B, the crude residue was purified by flash chromatography, using ethyl acetate:petroleum ether 0.5:9.5 (v:v) as eluent, to furnish **5p** as a colorless oil. Yield: 68%. ^1^H-NMR (CDCl_3_) δ: 1.36 (t, *J* = 7.0 Hz, 3H), 1.78–1.82 (m, 1H), 2.00–2.05 (m, 1H), 2.76–2.80 (m, 3H), 2.97–3.02 (m, 2H), 4.33 (q, *J* = 7.0 Hz, 2H), 7.24–7.33 (m, 5H). MS (ESI): [M]^+^ = 365.4, [M + 2]^+^ = 367.4.

#### 4.1.4. General Procedure C for the Preparation of Compounds (**3a**–**3p**)

A dry Schlenk tube was charged with dry toluene (5 mL), 2-bromo derivative **3a**–**p** (0.5 mmol), Pd(OAc)_2_ (3 mol%, 15 mg), *rac*-BINAP (4 mol%, 15 mg), Cs_2_CO_3_ (230 mg, 0.7 mmol, 1.4 equiv.), and 3,4,5-trimethoxyaniline (137 mg, 0.75 mmol, 1.5 equiv.) under argon, and the mixture was heated at 100 °C for 18 h. After cooling, the mixture was filtered on a pad of celite and the filtrate diluted with EtOAc (10 mL) and water (5 mL). The organic phase was washed with brine (5 mL), dried over Na_2_SO_4_, and concentrated under vacuum. The residue was purified by column chromatography on silica gel.

*Methyl 3-cyano-2-((3,4,5-trimethoxyphenyl)amino)-4,5-dihydrothieno[2,3-c]pyridine-6(7H)-carboxylate (**3a**).* Following general procedure C, the crude residue was purified by flash chromatography, using ethyl acetate:petroleum ether 3:7 (*v*:*v*) as eluent, to furnish **3a** as a yellow solid. Yield: 58%, m.p. 180–181 °C. ^1^H-NMR (*d_6_*-DMSO) δ: 2.50–2.53 (m, 2H), 3.59 (s, 3H), 3.62 (t, *J* = 5.8 Hz, 2H), 3.69 (s, 3H), 3.72 (s, 6H), 4.39 (s, 2H), 6.53 (s, 2H), 9.55 (s, 1H). ^13^C-NMR (*d_6_*-DMSO) δ: 23.92, 30.58, 42.31, 52.54, 55.66 (2C), 60.02, 90.03, 95.76 (2C), 114.79, 117.84, 130.87, 132.83, 137.99, 153.22 (2C), 155.41, 157.49. MS (ESI): [M + 1]^+^ = 404.3. Anal. calcd for C_19_H_21_N_3_O_5_S: C, 56.56; H, 5.25; N, 10.42; found: 56.38; H, 5.11; N, 10.21.

*Ethyl 3-cyano-2-((3,4,5-trimethoxyphenyl)amino)-4,5-dihydrothieno[2,3-c]pyridine-6(7H)-carboxylate* (**3b**). Following general procedure C, the crude residue was purified by flash chromatography, using ethyl acetate:petroleum ether 4:6 (*v*:*v*) as eluent, to furnish **3b** as a yellow solid. Yield: 51%, m.p. 134–136 °C. ^1^H-NMR (*d_6_*-DMSO) δ: 1.15 (t, *J* = 7.2 Hz, 3H), 2.50-2.53 (m, 2H), 3.59 (s, 3H), 3.63 (t, *J* = 5.8 Hz, 2H), 3.72 (s, 6H), 4.05 (q, *J* = 7.2 Hz, 2H), 4.39 (s, 2H), 6.53 (s, 2H), 9.55 (s, 1H). ^13^C-NMR (*d_6_*-DMSO) δ: 14.46, 23.92, 42.22, 55.67 (2C), 60.02, 61.02, 61.18, 95.74 (2C), 105.76, 114.80, 117.35, 130.89, 132.82, 137.99, 153.22 (2C), 154.62, 157.47. MS (ESI): [M + 1]^+^ = 418.4. Anal. calcd for C_20_H_23_N_3_O_5_S: C, 57.54; H, 5.55; N, 10.07; found: C, 57.32; H, 5.38; N, 9.98.

*Dimethyl 2-((3,4,5-trimethoxyphenyl)amino)-4,5-dihydrothieno[2,3-c]pyridine-3,6(7H)-dicarboxylate* (**3c**). Following general procedure C, the crude residue was purified by flash chromatography, using ethyl acetate:petroleum ether 4:6 (*v*:*v*) as eluent, to furnish **3c** as a white solid. Yield: 58%, m.p. 140–141 °C. ^1^H-NMR (*d_6_*-DMSO) δ: 2.75 (t, *J* = 5.6 Hz, 2H), 3.59 (t, *J* = 5.6 Hz, 2H), 3.63 (s, 6H), 3.78 (s, 9H), 4.39 (s, 2H), 6.68 (s, 2H), 9.62 (s, 1H). ^13^C-NMR (*d_6_*-DMSO) δ: 14.52, 26.24, 42.82, 51.51, 56.38 (2C), 60.20, 60.57, 97.92 (2C), 105.92, 114.33, 131.23, 134.09, 137.31, 153.86 (2C), 155.65, 158.70, 165.79. MS (ESI): [M + 1]^+^ = 437.5. Anal. calcd for C_20_H_24_N_2_O_7_S: C, 55.03; H, 5.54; N, 6.42; found: C, 54.89; H, 5.23; N, 6.30.

*6-Ethyl 3-methyl 2-((3,4,5-trimethoxyphenyl)amino)-4,5-dihydrothieno[2,3-c]pyridine-3,6(7H)-dicarboxylate* (**3d**). Following general procedure C, the crude residue was purified by flash chromatography, using ethyl acetate:petroleum ether 4:6 (*v*:*v*) as eluent, to furnish **3d** as a yellow solid. Yield: 55%, m.p. 76–78 °C. ^1^H-NMR (*d_6_*-DMSO) δ: 1.19 (t, *J* = 6.8 Hz, 3H), 2.82 (t, *J* = 5.8 Hz, 2H), 3.62 (t, *J* = 5.8 Hz, 2H), 3.64 (s, 3H), 3.77 (s, 3H), 3.78 (s, 6H), 4.06 (q, *J* = 6.8 Hz, 2H), 4.39 (s, 2H), 6.68 (s, 2H), 9.64 (s, 1H). ^13^C-NMR (*d_6_*-DMSO) δ: 14.58, 26.51, 42.30, 51.08, 55.94 (2C), 59.77, 60.14, 61.00, 97.43 (2C), 105.48, 114.32, 130.80, 133.64, 136.87, 153.42 (2C), 155.02, 158.24, 165.37. MS (ESI): [M + 1]^+^ = 451.5. Anal. (C_21_H_26_N_2_O_7_S) C, H, N. Anal. calcd for C_21_H_26_N_2_O_7_S. C, 55.99; H, 5.82; N, 6.22; found: C, 55.78; H, 5.64; N, 6.10.

*Methyl 6-acetyl-2-((3,4,5-trimethoxyphenyl)amino)-4,5,6,7-tetrahydrothieno[2,3-c]pyridine-3-carboxylate* (**3e**). Following general procedure C, the crude residue was purified by flash chromatography, using ethyl acetate:petroleum ether 1:9 (*v*:*v*) as eluent, to furnish **3e** as a yellow solid. Yield: 54%, m.p. 90–91 °C. ^1^H-NMR (*d_6_*-DMSO) δ: 2.06 (s, 3H), 2.79-2.82 (m, 2H), 3.61-3.64 (m, 5H), 3.76 (s, 9H), 4.42 (s, 2H), 6.66 (s, 2H), 9.61 (s, 1H). ^13^C-NMR (*d_6_*-DMSO) δ: 20.98, 27.09, 42.94, 43.98, 50.97, 55.83 (2C), 60.02, 97.31 (2C), 105.38, 114.08, 130.65, 133.52, 136.76, 153.31 (2C), 158.02, 165.22, 168.33. MS (ESI): [M + 1]^+^ = 421.3. Anal. calcd for C_20_H_24_N_2_O_6_S: C, 57.13; H, 5.75; N, 6.66; found: 57.01; H, 5.58; N, 6.48.

*3-Ethyl 6-methyl 2-((3,4,5-trimethoxyphenyl)amino)-4,5-dihydrothieno[2,3-c]pyridine-3,6(7H)-dicarboxylate* (**3f**). Following general procedure C, the crude residue was purified by flash chromatography, using ethyl acetate:petroleum ether 3:7 (*v*:*v*) as eluent, to furnish **3f** as a yellow solid. Yield: 59%, m.p. 128–130 °C. ^1^H-NMR (*d_6_*-DMSO) δ: 1.27 (t, *J* = 7.2 Hz, 3H), 2.73-2.75 (m, 2H), 3.52-3.54 (m, 2H), 3.58 (s, 6H), 3.79 (s, 6H), 4.21 (q, *J* = 7.2 Hz, 2H), 4.40 (s, 2H), 6.65 (s, 2H), 9.69 (s, 1H). ^13^C-NMR (*d_6_*-DMSO) δ: 14.21, 26.63, 42.41, 52.57, 55.09, 55.94 (2C), 59.72, 60.13, 97.17 (2C), 105.63, 114.55, 130.71, 133.53, 136.82, 153.44 (2C), 155.12, 158.07, 165.10. MS (ESI): [M + 1]^+^ = 451.6. Anal. calcd for C_21_H_26_N_2_O_7_S: C, 55.99; H, 5.82; N, 6.22; found: 55.77; H, 5.63; N, 6.02.

*Diethyl 2-((3,4,5-trimethoxyphenyl)amino)-4,5-dihydrothieno[2,3-c]pyridine-3,6(7H)-dicarboxylate* (**3g**). Following general procedure C, the crude residue was purified by flash chromatography, using ethyl acetate:petroleum ether 3:7 (*v*:*v*) as eluent, to furnish **3g** as a yellow solid. Yield: 63%, m.p. 67–69 °C. ^1^H-NMR (*d_6_*-DMSO) δ: 1.18 (t, *J* = 7.2 Hz, 3H), 1.29 (t, *J* = 7.2 Hz, 3H), 2.77 (t, *J* = 5.6 Hz, 2H), 3.62 (t, *J* = 5.6 Hz, 2H), 3.64 (s, 3H), 3.78 (s, 6H), 4.06 (q, *J* = 7.2 Hz, 2H), 4.23 (q, *J* = 7.2 Hz, 2H), 4.39 (s, 2H), 6.67 (s, 2H), 9.73 (s, 1H). ^13^C-NMR (*d_6_*-DMSO) δ: 14.20, 14.58, 26.33, 30.69, 42.31, 55.94, 59.72 (2C), 60.13, 61.00, 97.13 (2C), 105.62, 114.29, 130.74, 133.53, 136.81, 153.44 (2C), 154.67, 158.06, 165.12. MS (ESI): [M + 1]^+^ = 465.6. Anal. (C_22_H_28_N_2_O_7_S) C, H, N. Anal. calcd for C_22_H_28_N_2_O_7_S: C, 56.88; H, 6.08; N, 6.03; found: C, 56.65; H, 5.92; N, 5.88.

*Ethyl 6-acetyl-2-((3,4,5-trimethoxyphenyl)amino)-4,5,6,7-tetrahydrothieno[2,3-c]pyridine-3-carboxylate* (**3h**). Following general procedure C, the crude residue was purified by flash chromatography, using ethyl acetate:petroleum ether 1:9 (*v*:*v*) as eluent, to furnish **3h** as a white solid. Yield: 54%, m.p. 93-95 °C. ^1^H-NMR (*d_6_*-DMSO) δ: 1.28 (t, *J* = 7.2 Hz, 3H), 2.09 (s, 3H), 2.78-2.80 (m, 2H), 3.63 (s, 3H), 3.64-3.66 (m, 2H), 3.78 (s, 6H), 4.26 (q, *J* = 7.2 Hz, 2H), 4.45 (s, 2H), 6.67 (s, 2H), 9.72 (s, 1H). ^13^C-NMR (*d_6_*-DMSO) δ: 14.14, 20.99, 27.14, 42.97, 44.05, 55.83 (2C), 59.60, 60.02, 97.08 (2C), 105.54, 114.56, 130.59, 133.41, 136.71, 153.33 (2C), 157.97, 164.99, 168.33. MS (ESI): [M + 1]^+^ = 435.4. Anal. calcd for C_21_H_26_N_2_O_6_S: C, 62.20; H, 6.71; N, 3.45; found: C, 62.04; H, 6.58; N, 3.23.

*3-tert-butyl 6-methyl 2-((3,4,5-trimethoxyphenyl)amino)-4,5-dihydrothieno[2,3-c]pyridine-3,6(7H)-dicarboxylate* (**3i**). Following general procedure C, the crude residue was purified by flash chromatography, using ethyl acetate:petroleum ether 2:8 (*v*:*v*) as eluent, to furnish **3i** as a white solid. Yield: 64%, m.p. 50–52 °C. ^1^H-NMR (*d_6_*-DMSO) δ: 1.53 (s, 9H), 2.72–2.75 (m, 2H), 3.59 (t, *J* = 5.8 Hz, 2H), 3.63 (s, 3H), 3.76 (s, 3H), 3.78 (s, 6H), 4.40 (s, 2H), 6.67 (s, 2H), 9.74 (s, 1H). ^13^C-NMR (*d_6_*-DMSO) δ: 26.78, 27.98 (3C), 42.35, 52.47, 55.86 (2C), 59.67, 60.05, 80.50, 96.83 (2C), 106.89, 113.97, 130.67, 133.32, 136.74, 153.35 (2C), 155.02, 157.42, 164.75. MS (ESI): [M + 1]^+^ = 479.5. Anal. calcd for C_23_H_30_N_2_O_7_S: C, 57.72; H, 6.32; N, 5.85; found: C, 57.54; H, 6.21; N, 5.67.

*3-tert-butyl 6-ethyl 2-((3,4,5-trimethoxyphenyl)amino)-4,5-dihydrothieno[2,3-c]pyridine-3,6(7H)-dicarboxylate* (**3j**). Following general procedure C, the crude residue was purified by flash chromatography, using ethyl acetate:petroleum ether 3:7 (*v*:*v*) as eluent, to furnish **3j** as a yellow solid. Yield: 70%, m.p. 60–62 °C. ^1^H-NMR (*d_6_*-DMSO) δ: 1.17 (t, *J* = 7.2 Hz, 3H), 1.53 (s, 9H), 2.74 (t, *J* = 5.8 Hz, 2H), 3.59 (t, *J* = 5.8 Hz, 2H), 3.63 (s, 3H), 3.78 (s, 6H), 4.06 (q, *J* = 7.2 Hz, 2H), 4.39 (s, 2H), 6.67 (s, 2H), 9.75 (s, 1H). ^13^C-NMR (*d_6_*-DMSO) δ: 15.04, 24.02, 28.50 (3C), 31.14, 42.77, 56.38 (2C), 60.57, 61.44, 81.03, 97.32 (2C), 107.39, 114.04, 131.42, 133.83, 137.26, 153.88 (2C), 155.31, 157.93, 165.30. MS (ESI): [M + 1]^+^ = 493.4. Anal. calcd for C_24_H_32_N_2_O_7_S: C, 58.52; H, 6.55; N, 5.69; found: C, 58.29; H, 6.29; N, 5.41.

*6. -Methyl-2-((3,4,5-trimethoxyphenyl)amino)-4,5,6,7-tetrahydrobenzo[b]thiophene-3-carbonitrile* (**3k**). Following general procedure C, the crude residue was purified by flash chromatography, using ethyl acetate:petroleum ether 2:8 (*v*:*v*) as eluent, to furnish **3k** as a white solid. Yield: 58%, m.p. 112–114 °C. ^1^H-NMR (*d_6_*-DMSO) δ: 1.01 (d, *J* = 6.4 Hz, 3H), 1.37–1.40 (m, 1H), 1.80–1.82 (m, 2H), 2.14–2.18 (m, 1H), 2.50–2.52 (m, 2H), 2.61–2.65 (m, 1H), 3.61 (s, 3H), 3.73 (s, 6H), 6.52 (s, 2H), 9.41 (s, 1H). ^13^C-NMR (*d_6_*-DMSO) δ: 21.61, 24.14, 29.62, 30.31, 32.19, 56.17 (2C), 60.57, 91.43, 95.78 (2C), 115.76, 121.73, 132.12, 133.00, 138.88, 153.74 (2C), 156.67. MS (ESI): [M + 1]^+^ = 359.4. Anal. calcd for C_19_H_22_N_2_O_5_S: C, 63.66; H, 6.19; N, 7.82; found: C, 63.47; H, 6.01; N, 7.64.

*6. -Phenyl-2-((3,4,5-trimethoxyphenyl)amino)-4,5,6,7-tetrahydrobenzo[b]thiophene-3-carbonitrile* (**3l**). Following general procedure C, the crude residue was purified by flash chromatography, using ethyl acetate:petroleum ether 2:8 (*v*:*v*) as eluent, to furnish **3l** as a yellow solid. Yield: 63%, m.p. 169–170 °C. ^1^H-NMR (*d_6_*-DMSO) δ: 1.96–1.99 (m, 2H), 2.60–2.63 (m, 3H), 2.74–2.78 (m, 1H), 2.98–3.01 (m, 1H), 3.61 (s, 3H), 3.74 (s, 6H), 6.54 (s, 2H), 7.20–7.23 (m, 1H), 7.30-7.32 (m, 4H), 9.48 (s, 1H). ^13^C-NMR (*d_6_*-DMSO) δ: 24.80, 29.43, 32.02, 40.02, 56.19 (2C), 60.58, 91.25, 95.92 (2C), 115.75, 121.70, 126.80, 126.32 (2C), 128.87 (2C), 132.22, 133.10, 138.82, 145.91, 153.75 (2C), 156.99. MS (ESI): [M + 1]^+^ = 421.5. Anal. (C_24_H_24_N_2_O_5_S) C, H, N. Anal. calcd for C_24_H_24_N_2_O_5_S: C, 68.55; H, 5.75; N, 6.66; found: C, 68.34; H, 5.54; N, 6.26.

*Methyl 6-methyl-2-((3,4,5-trimethoxyphenyl)amino)-4,5,6,7-tetrahydrobenzo[b]thiophene-3-carboxylate* (**3m**). Following general procedure C, the crude residue was purified by flash chromatography, using ethyl acetate:petroleum ether 2:8 (*v*:*v*) as eluent, to furnish **3m** as a white solid. Yield: 55%, m.p. 127-129 °C. ^1^H-NMR (*d_6_*-DMSO) δ: 0.99 (d, *J* = 6.8 Hz, 3H), 1.26-1.30 (m, 1H), 1.76–1.80 (m, 2H), 2.13–2.16 (m, 1H), 2.50–2.54 (m, 2H), 2.82- 2.84 (m, 1H), 3.63 (s, 3H), 3.76 (s, 3H), 3.77 (s, 6H), 6.65 (s, 2H), 9.70 (s, 1H). ^13^C-NMR (*d_6_*-DMSO) δ: 21.67, 26.50, 29.30, 30.93, 32.36, 51.40, 56.35 (2C), 60.57, 97.35 (2C), 106.39, 117.77, 131.88, 133.74, 137.47, 153.83 (2C), 157.61, 166.18. MS (ESI): [M + 1]^+^ = 392.3. Anal. (C_20_H_25_NO_5_S) C, H, N. Anal. calcd for C_24_H_24_N_2_O_5_S: C, 61.36; H, 6.44; N, 3.58; found: C, 61.18; H, 6.23; N, 3.41.

*Methyl 6-phenyl-2-((3,4,5-trimethoxyphenyl)amino)-4,5,6,7-tetrahydrobenzo[b]thiophene-3-carboxylate* (**3n**). Following general procedure C, the crude residue was purified by flash chromatography, using ethyl acetate:petroleum ether 2:8 (*v*:*v*) as eluent, to furnish **3n** as a white solid. Yield: 61%, m.p. 147-148 °C. ^1^H-NMR (*d_6_*-DMSO) δ: 1.79–1.82 (m, 1H), 1.96–2.00 (m, 1H), 2.68–2.72 (m, 3H), 2.83–2.86 (m, 2H), 3.62 (s, 3H), 3.78 (s, 9H), 6.68 (s, 2H), 7.22 (m, 1H), 7.31 (m, 4H), 9.71 (s, 1H). ^13^C-NMR (*d_6_*-DMSO) δ: 26.59, 29.47, 31.52, 40.02, 50.90, 55.82 (2C), 60.02, 97.00 (2C), 105.75, 117.27, 126.11, 126.73 (2C), 128.26 (2C), 131.49, 133.31, 136.90, 145.63, 153.29 (2C), 157.35, 165.60. MS (ESI): [M + 1]^+^ = 454.4. Anal. calcd for C_25_H_27_NO_5_S: C, 66.20; H, 6.00; N, 3.09; found: C, 66.01; H, 5.84; N, 2.92.

*Ethyl 6-methyl-2-((3,4,5-trimethoxyphenyl)amino)-4,5,6,7-tetrahydrobenzo[b]thiophene-3-carboxylate* (**3o**). Following general procedure C, the crude residue was purified by flash chromatography, using ethyl acetate:petroleum ether 2:8 (*v*:*v*) as eluent, to furnish **3o** as a white solid. Yield: 56%, m.p. 90–92 °C. ^1^H-NMR (*d_6_*-DMSO) δ: 0.99 (d, *J* = 6.4 Hz, 3H), 1.26–1.28 (m, 1H), 1.32 (t, *J* = 7.0 Hz, 3H), 1.79–1.82 (m, 2H), 2.16–2.20 (m, 1H), 2.52–2.58 (m, 2H), 2.79-2.82 (m, 1H), 3.62 (s, 3H), 3.77 (s, 6H), 4.24 (q, *J* = 7.0 Hz, 2H), 6.64 (s, 2H), 9.79 (s, 1H). ^13^C-NMR (*d_6_*-DMSO) δ: 14.15, 21.10, 26.01, 28.73, 30.43, 31.83, 55.80 (2C), 59.44, 60.02, 96.54 (2C), 105.98, 117.26, 131.29, 133.10, 136.88, 153.31 (2C), 156.89, 165.38. MS (ESI): [M + 1]^+^ = 406.3. Anal. calcd for C_21_H_27_NO_5_S: C, 62.20; H, 6.71; N, 3.45; found: C, 62.01; H, 6.48; N, 3.23.

*Ethyl 6-phenyl-2-((3,4,5-trimethoxyphenyl)amino)-4,5,6,7-tetrahydrobenzo[b]thiophene-3-carboxylate* (**3p**). Following general procedure C, the crude residue was purified by flash chromatography, using ethyl acetate:petroleum ether 2:8 (*v*:*v*) as eluent, to furnish **3p** as a yellow solid. Yield: 73%, m.p. 98-100 °C. ^1^H-NMR (*d_6_*-DMSO) δ: 1.19 (t, *J* = 6.8 Hz, 3H), 1.84-1.86 (m, 1H), 1.98–2.02 (m, 1H), 2.79–2.84 (m, 3H), 2.97–3.00 (m, 2H), 3.60 (s, 3H), 3.82 (s, 6H), 4.28 (q, *J* = 7.0 Hz, 2H), 6.67 (s, 2H), 7.19–7.21 (m, 1H), 7.29–7.33 (m, 4H), 9.80 (s, 1H). ^13^C-NMR (*d_6_*-DMSO) δ: 14.17, 26.65, 29.57, 31.49, 55.82 (2C), 59.42, 59.65, 60.02, 96.72 (2C), 105.90, 117.32, 126.13, 126.73 (2C), 128,27 (2C), 131.44, 133.20, 136.86, 145.65, 153.31 (2C), 157.19, 165.35. MS (ESI): [M + 1]^+^ = 468.4. Anal. calcd for C_26_H_29_NO_5_S: C, 66.79; H, 6.25; N, 3.00; found: C, 66.58; H, 6.02; N, 2.78.

### 4.2. Biological Evaluation

#### 4.2.1. Cell Growth Conditions and Antiproliferative Assay

Murine leukemia L1210, human T-lymphocyte leukemia CEM, human cervix carcinoma (HeLa), and human chronic myelogenous leukemia K562 cells (3–5 × 10^4^ cells) and a serial (5-fold) dilution of the test compounds were added to a 96-well microtiter plate. The cells were incubated for 72 h at 37 °C in a humidified 5% CO_2_ atmosphere. At the end of the incubation period, the cells were counted in a Coulter Counter (Coulter Electronics Ltd., Harpenden Herts, United Kingdom). The IC_50_ (50% inhibitory concentration) was defined as the concentration of compound that inhibited cell proliferation by 50%. The IC_50_ values represent the average (± standard deviation) of three independent experiments. Human peripheral blood mononuclear cells (PBMC) were obtained from healthy donors by centrifugation with Ficoll-Paque Plus (GE Healthcare Bio-Sciences AB, Uppsala).

#### 4.2.2. Effects on Tubulin Polymerization

Bovine brain tubulin was purified as described previously [58]. To evaluate the effect of the compounds on tubulin assembly in vitro [59]*,* varying concentrations were preincubated with 10 μM tubulin in 0.8 M glutamate buffer at 30 °C and then cooled to 0 °C. After addition of GTP, the mixtures were transferred to 0 °C cuvettes in a recording spectrophotometer and warmed to 30 °C, and the assembly of tubulin was observed turbidimetrically. The IC_50_ was defined as the compound concentration that inhibited the extent of assembly by 50% after a 20 min incubation. The ability of the test compounds to inhibit colchicine binding to tubulin was measured as described earlier [60], except that the reaction mixtures contained 1 μM tubulin, 5 μM of [^3^H]colchicine, and test compounds **3a** or **3b**.

#### 4.2.3. Effects on the Cell Cycle

Human leukemia K562 cells were treated with compounds **3a** or **3b**, and analysis of the distribution of the cells through the cell cycle was performed with the Muse Cell Analyzer instrument (Millipore Corporation, Billerica, MA, USA). Assay and cell counting were performed according to the instructions supplied by the manufacturer. Samples were collected after the treatment and washed in PBS. For analysis, 5 × 10^5^ cells were resuspended in 200 μL of Muse Cell Cycle Reagent, incubated for 30 min at room temperature, and the cell suspension was transferred to a 1.5 mL microcentrifuge tube prior to analysis. Cells were analyzed by fluorescence-activated cell sorting analysis using a Muse Cell Analyzer.

#### 4.2.4. Effects on Apoptosis

Apoptosis assays on K562 cells were performed with the Muse Cell Analyzer instrument, and assays were performed according to the instructions supplied by the manufacturer. The Muse Annexin V & Dead Cell Kit employs annexin V to detect phosphatidyl serine (PS) on the external membrane of apoptotic cells. A fluorescent DNA intercalator (7-ADD: 7-aminoactinomycin D) is also used as an indicator of cell membrane integrity. From live, healthy cells, as well as early apoptotic cells, 7-ADD is excluded while it is able to bind DNA in cells in late apoptosis and in dead cells. Cells were washed with sterile PBS 1X, suspended, and diluted (1:2) with the Muse Annexin V & Dead Cell reagent. Samples were gently mixed and incubated at room temperature, protected from the light for 15 min. Samples were analyzed using a Muse Cell Analyzer, and data from prepared samples were acquired and recorded utilizing the Annexin V and Dead Cell Software Module (Millipore) [61].

#### 4.2.5. Molecular Modeling Methods

All molecular docking studies were performed on a Viglen Genie Intel^®^Core^TM^ i7-3770 vPro CPU@ 3.40 GHz x 8 running Ubuntu 18.04. Molecular Operating Environment (MOE) 2019.10 [62] and Maestro (Schrödinger Release 2019-3) [54] were used as molecular modeling software. The tubulin structure was downloaded from the PDB data bank (http://www.rcsb.org/; PDB code 4O2B). The protein was preprocessed using the Schrödinger Protein Preparation Wizard by assigning bond orders, adding hydrogens and performing a restrained energy minimization of the added hydrogens using the OPLS_2005 force field. Ligand structures were built with MOE and then prepared using the Maestro LigPrep tool by energy minimizing the structures (OPLS_2005 force field), generating possible ionization states at pH 7 ± 2, generating tautomers and low-energy ring conformers. After isolating a tubulin dimer structure, a 12 Å docking grid (inner-box 10 Å and outer-box 22 Å) was prepared using as centroid the co-crystallized colchicine. Molecular docking studies were performed using Glide SP precision, keeping the default parameters and setting 5 as the number of output poses per input ligand to include in the solution. The output database was saved as a mol2 file. The docking results were visually inspected for their ability to bind the active site.

## 5. Conclusions

In conclusion, we have described the synthesis and biological evaluation of two classes of compounds based on the 4,5,6,7-tetrahydrothieno[2,3-*c*]pyridine and 4,5,6,7-tetrahydrobenzo[*b*]thiophene skeletons, both characterized by the presence of a common 3′,4′,5′-trimethoxyanilino moiety at the 2-position and a cyano or different alkoxycarbonyl groups at the 3-position. In the series of 2-(3′,4′,5′-trimethoxyanilino)-4,5,6,7-tetrahydrothieno[2,3-*c*]pyridine derivatives, the results indicated that inhibition of cell growth was strongly dependent on the substituent at the 3-position, with the greatest activity occurring with the cyano group, the least with alkoxycarbonyl moieties such as methoxy/ethoxy/*tert*-butoxycarbonyl. We also demonstrated that replacement of the 4,5,6,7-tetrahydrothieno[2,3-c]pyridine moiety with a 4,5,6,7-tetrahydrobenzo[*b*]thiophene system was detrimental for antiproliferative activity. The 2-(3′,4′,5′-trimethoxyanilino)-3-cyano-6-methoxycarbonyl-4,5,6,7-tetrahydrothieno[2,3-*c*]pyridine derivative **3a** and the corresponding 6-ethoxycarbonyl homologue **3b** were the most promising compounds in this series, which inhibited cancer cell growth at low micromolar concentrations and interacted with tubulin by binding to the colchicine site. Comparing the two 4,5,6,7-tetrahydrothieno[2,3-*c*]pyridine-3-carbonitrile derivatives **3a** and **3b**, the latter was about 1.5-fold more active than **3a** against L1210 and HeLa cells, while both these derivatives were equipotent against CEM cells. We also showed by flow cytometry that derivatives **3a** and **3b** had cellular effects typical of agents that bind to tubulin, causing accumulation of cells in the G2-M phase of the cell cycle. Apoptotic cells also increased in a concentration-dependent manner, with an increase in the percentage of apoptotic K562 cells observed at the IC_50_ values examined for **3a** and **3b** (0.75 and 0.70 μM, respectively). Finally, the same compounds showed high sensitivity towards cancer over normal cells as they had no significant antiproliferative activity toward both quiescent and phytohemoagglutinin-stimulated cultures of PBMC (IC_50_ > 20 μM), suggesting that derivatives **3a** and **3b** may have selectivity against cancer cells.

## Figures and Tables

**Table 1:** In vitro growth inhibition activity of compounds **3a**–**3p** and reference compounds **2b** and CA-4 against the proliferation of murine leukemia (L1210), human T-lymphocyte (CEM), and human cervix carcinoma (HeLa) cells.

IC_50_ (μM) ^a^			
Compound	L1210	CEM	HeLa
**3a**	4.7 ± 0.4	2.9 ± 2.1	1.9 ± 1.2
**3b**	2.8 ± 2.3	2.3 ± 1.7	1.1 ± 0.0
**3c**	17 ± 5	16 ± 4	23 ± 10
**3d**	15 ± 2	12 ± 4	16 ± 3
**3e**	26 ± 2	43 ± 12	48 ± 17
**3f**	22 ± 4	22 ± 8	67 ± 20
**3g**	19 ± 0	19 ± 1	19 ± 4
**3h**	25 ± 3	34 ± 9	24 ± 3
**3i**	18 ± 3	20 ± 2	14 ± 1
**3j**	19 ± 3	19 ± 2	20 ± 4
**3k**	>250	>250	202 ± 25
**3l**	92 ± 29	58 ± 53	124 ± 8
**3m**	82 ± 13	45 ± 17	11 ± 6
**3n**	101 ± 9	102 ± 50	48 ± 6
**3o**	86 ± 41	78 ± 33	66 ± 6
**3p**	112 ± 1	88 ± 12	169 ± 18
**2b**	6.0 ± 0.3	3.7 ± 1.2	41 ± 27
**CA-4 (nM)**	2.8 ± 1.1	1.9 ± 1.1	4.1 ± 0.1

^a^ IC_50_= compound concentration required to inhibit tumor cell proliferation by 50%. Data are expressed as the mean ± SE from the dose-response curves of at least three independent experiments.

**Table 2:** Inhibition of tubulin polymerization and colchicine binding by compounds **3a**, **3b**, and CA-4.

Compound	Tubulin assembly ^a^ IC_50_ ± SD (μM)	Colchicine binding ^b^ % ± SD
**3a**	3.8 ± 0.05	29 ± 1
**3b**	3.4 ± 0.05	31 ± 1
**CA-4**	0.54 ± 0.06	98 ± 0.9

^a^ Inhibition of tubulin polymerization. Tubulin was at 10 μM. ^b^ Inhibition of [^3^H]colchicine binding. Tubulin, colchicine, and tested compound were at 1, 5, and 5 μM, respectively.
